# Ustilago

**Published:** 1888-04

**Authors:** L. Hamilton Evans


### USTILAGO.

In cases calling for this drug, the discharge is of mixed character, partly liquid, partly coagulated, in this respect resembling Sabina, rather than its congener, Secale, in which the blood is generally thin and liquid. The flow is passive, slow, and long continued. Similarly suggestive of atonic condition of the parts concerned, we find, among its symptoms, deficiency of labor pains, with dilatable os uteri. When we have added metrorrhagia with vertigo, during the climacteric period, and menorrhagia with displaced uterus, we shall have included the most important hemorrhagic symptoms in our description.

Among the general symptoms we find depression of spirits; partial loss of control over the functions of vision and deglutition; and, suggestive at last of some resemblance to Secale, in nature, as in origin, oppression and faintness in a warm room and aversion to warmth in general.

L. Hamilton Evans, M. D.

